# Protocol for a Group-Sequential Two-Stratum Multicenter Open-Label Randomized Clinical Trial of Respiratory Support in Infants With Acute Bronchiolitis: Breathing Assistance in Children With Bronchiolitis (BACHb)

**DOI:** 10.1097/PCC.0000000000003813

**Published:** 2025-08-14

**Authors:** Isabel Johnson, Katy Bridges, Richard Cleaver, Rayka Malek, Mary Cross, Steve Cunningham, Katrina Cathie, Mark D. Lyttle, Rebecca Mitting, Paul Mouncey, Damian Roland, Stephen Turner, Olu Onyimadu, Stavros Petrou, Debra Quantrill, Kate Chadwick, Leila Janani, Padmanabhan Ramnarayan

**Affiliations:** 1 Children’s Clinical Research Facility, Imperial College Healthcare NHS Trust, London, United Kingdom.; 2 Department of Surgery and Cancer, Imperial College London, London, United Kingdom.; 3 Imperial Clinical Trials Unit, School of Public Health, Imperial College London, London, United Kingdom.; 4 Department of Child Life and Health, Centre for Inflammation Research, University of Edinburgh, Edinburgh, United Kingdom.; 5 NIHR Southampton Clinical Research Facility and Biomedical Research Centre, University Hospital Southampton NHS Foundation Trust, Southampton, United Kingdom.; 6 Emergency Department, Bristol Royal Hospital for Children, Bristol, United Kingdom.; 7 Research in Emergency Care Avon Collaborative Hub, University of the West of England, Bristol, United Kingdom.; 8 Paediatric Intensive Care Unit, Imperial College Healthcare NHS Trust, London, United Kingdom.; 9 Intensive Care National Audit and Research Centre (ICNARC), London, United Kingdom.; 10 Paediatric Emergency Medicine Leicester Academic (PEMLA Group), Children’s Emergency Department, University Hospitals of Leicester, Leicester, United Kingdom.; 11 SAPPHIRE Group, Population Health Sciences, Leicester University, Leicester, United Kingdom.; 12 Women and Children Division, NHS Grampian, Aberdeen, United Kingdom.; 13 University of Oxford, Oxford, United Kingdom.; 14 Patient Representative, United Kingdom.; 15 Centre for Paediatrics and Child Health, Imperial College London, London, United Kingdom.

**Keywords:** bronchiolitis, continuous positive airway pressure, high-flow nasal cannula therapy, humidified oxygen, noninvasive respiratory support

## Abstract

**OBJECTIVES:**

The Breathing Assistance in Children with bronchiolitis (BACHb) trial aims to evaluate the clinical and cost-effectiveness of high-flow nasal cannula (HFNC) therapy compared with humidified standard oxygen (HSO) in infants with moderate bronchiolitis, and HFNC with continuous positive airway pressure (CPAP) in severe bronchiolitis.

**DESIGN:**

Pragmatic, group-sequential, two-stratum, multicenter, open-label randomized clinical trial.

**SETTING:**

Fifty hospitals across England, Scotland, and Wales.

**PATIENTS:**

Hospitalized infants younger than 12 months old with a clinical diagnosis of bronchiolitis, assessed at least twice 15 minutes apart to fulfill criteria for either severe bronchiolitis (one or more of: respiratory rate > 70 breaths/min, grunting, marked chest recession, recurrent short apneas) or moderate bronchiolitis (lack of response to low-flow oxygen, indicated by persistent hypoxemia and/or moderate respiratory distress).

**INTERVENTIONS:**

“Moderate bronchiolitis stratum”: HFNC at a flow rate of 2 L/kg/min vs. HSO through a facemask or headbox at a flow rate up to 15 L/min. “Severe bronchiolitis stratum”: HFNC at a flow rate of 2 L/kg/min vs. CPAP pressure set at 6–8 cm H_2_O.

**MEASUREMENTS AND MAIN RESULTS:**

In each stratum, eligible infants will be randomly allocated on a 1:1 basis to the trial treatments using a web-based system by permuted block randomization, stratified by site of recruitment and age (< 6 wk and ≥ 6 wk). Due to the emergency nature of the treatments, written informed consent will be deferred. The primary outcome is time from randomization to hospital discharge within 30 days. Baseline clinical characteristics and hospital course, including details of respiratory support, and discharge and cost-effectiveness outcomes will be collected. The trial received Health Research Authority and Research Ethics Committee approval from the Yorkshire and The Humber—South Yorkshire Research Ethics Committee on August 3, 2023 (reference: 23/YH/0166). The trial registration is ISRCTN52937119.

**CONCLUSIONS:**

Trial findings will be disseminated in national and international conferences, in peer-reviewed journals and through social media.

**RESEARCH IN CONTEXT**

- Bronchiolitis is a common reason for critical care admission, posing an enormous burden on hospitals worldwide, mainly during winter months, and is associated with considerable healthcare costs.
- Even though hypoxemia and breathing difficulties are the most important reasons for infants needing critical care admission, there are few well-powered randomized controlled trials comparing different modes of noninvasive respiratory support in infants with bronchiolitis-associated respiratory failure.
- The Breathing Assistance in Children with bronchiolitis (BACHb) trial is a group-sequential design, two-stratum, multicenter, open-label randomized clinical trial of respiratory support enrolling 1508 infants with acute bronchiolitis in 50 hospitals in the United Kingdom.

**AT THE BEDSIDE**

- The BACHb trial is the first large multicenter adaptive randomized controlled trial in acute bronchiolitis to compare different modes of noninvasive respiratory support in infants with moderate as well as severe bronchiolitis.
- The two-stratum design allows results to be generated independently in each stratum (moderate and severe bronchiolitis) and allow early stopping of either stratum for efficacy without affecting the rest of the trial.
- The BACHb trial has the potential to produce important research findings, inform clinical practice as well as health policy worldwide, and lead to improvements in outcomes and the healthcare experience of infants with bronchiolitis and their families.

Bronchiolitis results in the hospitalization of approximately 30,000 infants per year in England (1, 2). Nearly 1000 require admission to a PICU, mainly for invasive ventilation (3, 4). This burden exacerbates existing staff and critical care bed shortages and accounts for considerable healthcare costs, partly from overuse of possibly ineffective treatments (5–9). A core aspect of bronchiolitis management is oxygen administration for hypoxemia and respiratory support for breathing difficulties (10). Guidelines commonly recommend low-flow oxygen (LFO) as the first step for oxygen supplementation in infants with hypoxemia (11). In sicker infants; however, there is ongoing uncertainty regarding the most effective methods of respiratory support (12).

Despite lack of evidence from well-powered randomized controlled trials (RCTs), high-flow nasal cannula (HFNC) therapy is widely used as an alternative to continuous positive airway pressure (CPAP) in infants with impending respiratory failure (13–16). HFNC is also commonly started in infants who “fail” treatment with LFO, based on physiologic and observational studies suggesting improved patient comfort and ease of use (17–19). In these infants, alternative options such as humidified standard oxygen (HSO) may provide adequate humidification and could be delivered in a ward environment with a lower nurse-to-patient ratio of 1:4 (compared with 1:2 for HFNC), with lower hospitalization costs (20–22). However, there are few RCTs comparing these methods of respiratory support.

The Breathing Assistance in Children with bronchiolitis (BACHb) trial is a group-sequential design, two-stratum, multicenter, open-label randomized clinical trial of respiratory support in infants with acute bronchiolitis. The trial will test the hypothesis that the first-line use of HFNC is superior to HSO in infants with bronchiolitis who have “failed” LFO (moderate bronchiolitis stratum) and CPAP in infants with impeding respiratory failure (severe bronchiolitis stratum) in reducing the time to hospital discharge.

## METHODS

The main aim of BACHb is to evaluate the clinical and cost-effectiveness of HFNC compared with HSO (in infants with moderate bronchiolitis) and CPAP (in infants with severe bronchiolitis).

### Primary Objective

The primary objective is to evaluate the effectiveness of HFNC compared with HSO (moderate bronchiolitis) and CPAP (severe bronchiolitis) in reducing the time to hospital discharge.

### Design and Setting

The trial is a pragmatic (23), four-stage group-sequential design, two-stratum, multicenter, open-label RCT with integrated health economic evaluation. The two-stratum design allows each stratum to produce independent results, as well as enabling early stopping for efficacy without affecting the rest of the trial.

A 6-month internal pilot phase (from October 2023 to March 2024) assessed key progression criteria of patient recruitment, and number of sites open to recruitment (green criteria: ≥ 40 sites open to recruitment, ≥ 150 patients enrolled and a recruitment rate of at least 1.1 per site per month). The trial funder recommended progression from the pilot stage to the full trial, based upon the recommendation of the Trial Steering Committee (TSC).

Study recruitment will continue to occur across Emergency Departments, inpatient pediatric wards and assessment units, and Paediatric Critical Care Units across England, Scotland, and Wales. Sites will be identified through the Paediatric Emergency Research in the United Kingdom and Ireland (PERUKI) (24), General and Adolescent Paediatric Research in the United Kingdom and Ireland (GAPRUKI) (25), and Paediatric Critical Care Society Study Group (PCCS-SG) (26). Sites are capable of administering the required trial treatments (HSO, HFNC, and CPAP), have collective equipoise between all possible treatments and comply with all study requirements as per the study protocol in addition to complying with legal and ethical requirements of the U.K. Policy Framework for Health and Social Care Research and the International Conference on Harmonization Guidelines on Good Clinical Practice (ICH-GCP).

### Study Population and Screening

The study population will include hospitalized infants with acute bronchiolitis (**Table 1**). Eligible patients will be screened by clinical and research staff, for the severe stratum first, and if inclusion criteria are not met, for the moderate stratum. Fulfillment of any exclusion criteria will result in the patient being ineligible. Ineligible patients will be recorded on site screening and enrollment logs, explaining reasons for exclusion.

**TABLE 1.** Eligibility Criteria for the Breathing Assistance in Children With Bronchiolitis (BACHb) Trial

**Inclusion criteria (severe bronchiolitis**)
Hospitalized infants < 12 mo old with a clinical diagnosis of acute bronchiolitis, clinically assessed twice at least 15 min apart to have:
severe respiratory distress (one or more of: respiratory rate > 70 breaths/min, grunting, marked chest recession) and/or
recurrent short apneas (> 3/hr, each apnea lasting < 10 s)
**Inclusion criteria (moderate bronchiolitis**)
Hospitalized infants < 12 mo old with a clinical diagnosis of acute bronchiolitis, clinically assessed twice at least 15 min apart to have a lack of response to low-flow oxygen (up to 2 L/min), as indicated by:
persistent hypoxemia (oxygen saturation < 90%, or < 92% if age < 6 wk or if underlying health problems present) and/or
moderate respiratory distress (respiratory rate 55–70/min and/or moderate chest recession)
**Exclusion criteria**
1) Clinical decision that patient needs immediate intubation and ventilation for life-threatening hypoxemia, shock, or decreased conscious level
2) Prolonged apneas (> 10 s needing stimulation)
3) Ongoing active air leak (pneumothorax, pneumomediastinum)
4) Received humidified standard oxygen, high-flow nasal cannula, or continuous positive airway pressure for over 2 hr in the previous 24 hr
5) On home ventilation before hospital admission
6) Tracheostomy
7) Choanal atresia/stenosis, midfacial anomalies, or recent craniofacial surgery
8) Previously recruited to the Breathing Assistance in Children with bronchiolitis (BACHb) trial in the last 90 d

### Randomization and Concealment

Patients will be randomized as soon as possible following confirmation of eligibility to avoid delays in starting treatment. Randomization will occur at a 1:1 ratio using permuted blocks to either HFNC or HSO (moderate stratum) or HFNC or CPAP (severe stratum) using a web-based system (Sealed Envelope, London, United Kingdom), and stratified by the site of recruitment and infant’s age (< 6 and ≥ 6 wk). Once randomized into one stratum, patients cannot be randomized to the other stratum (e.g., from moderate to severe). The final randomization list was generated and stored by Sealed Envelope; none of the trial team members have access to the stored list, ensuring allocation concealment.

### Blinding

Since the medical devices and the interfaces that deliver the trial treatments are easily distinguishable from each other, it will not be possible to blind the subject or clinical staff.

### Study Treatments

#### Humidified Standard Oxygen

HSO will be delivered as per usual practice at each site using either a headbox or facemask attached to a heater/humidifier. The gas flow rate will be titrated between 6 and 15 L/min to maintain oxygen saturations greater than 90% (or > 92% for infants < 6 wk old or children of any age with underlying health conditions) (27).

#### High-Flow Nasal Cannula

HFNC will involve the delivery of heated, humidified gas through nasal cannula at the prescribed high-flow rate (2 L/kg/min). No specific device is recommended, and sites are expected to use their usual equipment.

#### Continuous Positive Airway Pressure

CPAP may be delivered using any medically approved device that can provide an expiratory pressure of 6–8 cm H_2_O that is used as part of the site’s usual practice.

### Clinical Practice During the Trial

**Figure 1.**
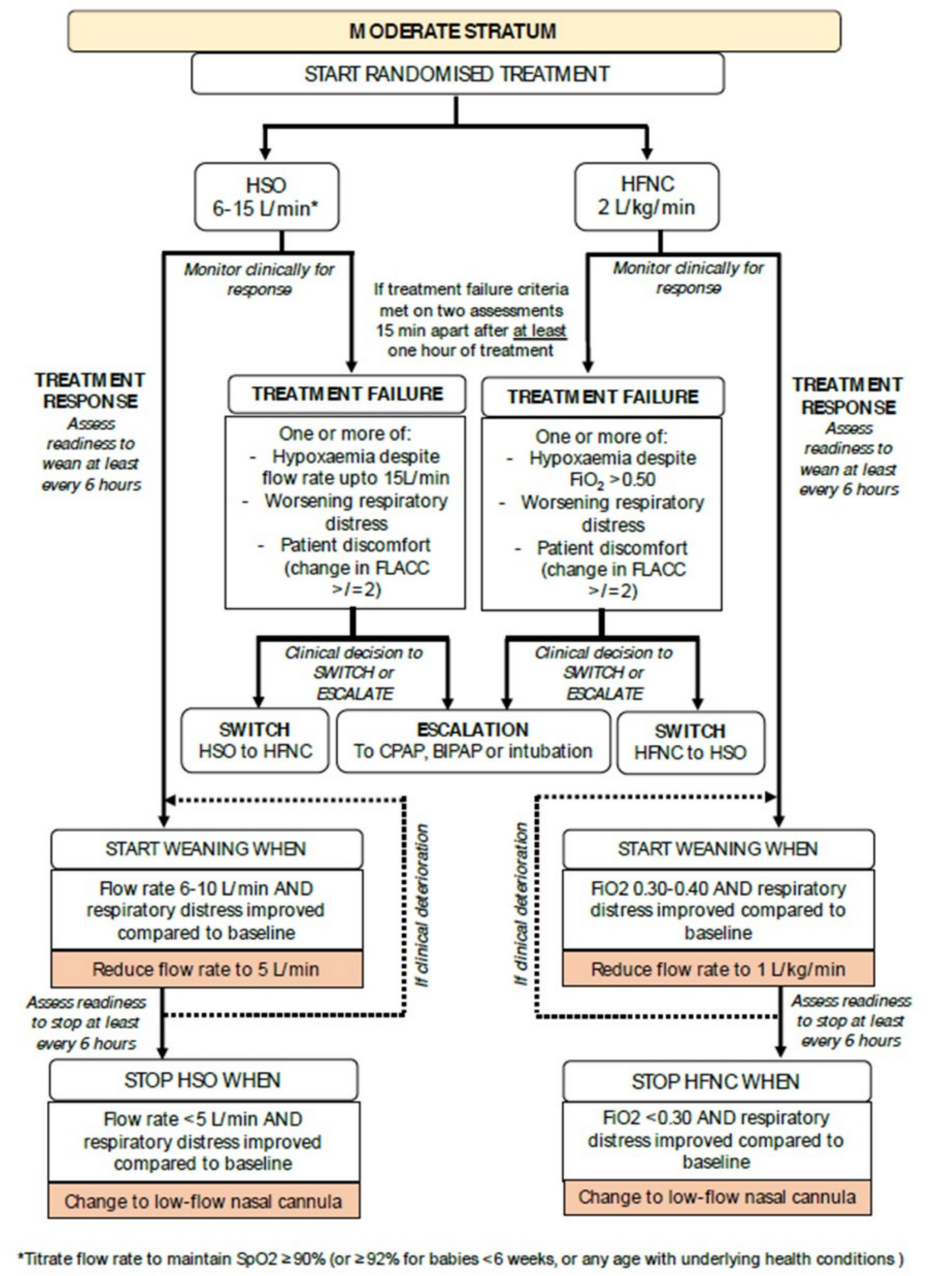
Algorithm for the initiation, weaning, switching, and escalation of treatment for the moderate stratum. BIPAP = bilevel positive pressure ventilation, CPAP = continuous positive airway pressure, FLACC = Face, Legs, Activity, Crying, and Consolability, HFNC = high-flow nasal cannula, HSO = humidified standard oxygen, Spo_2_ = oxygen saturation.

**Figure 2.**
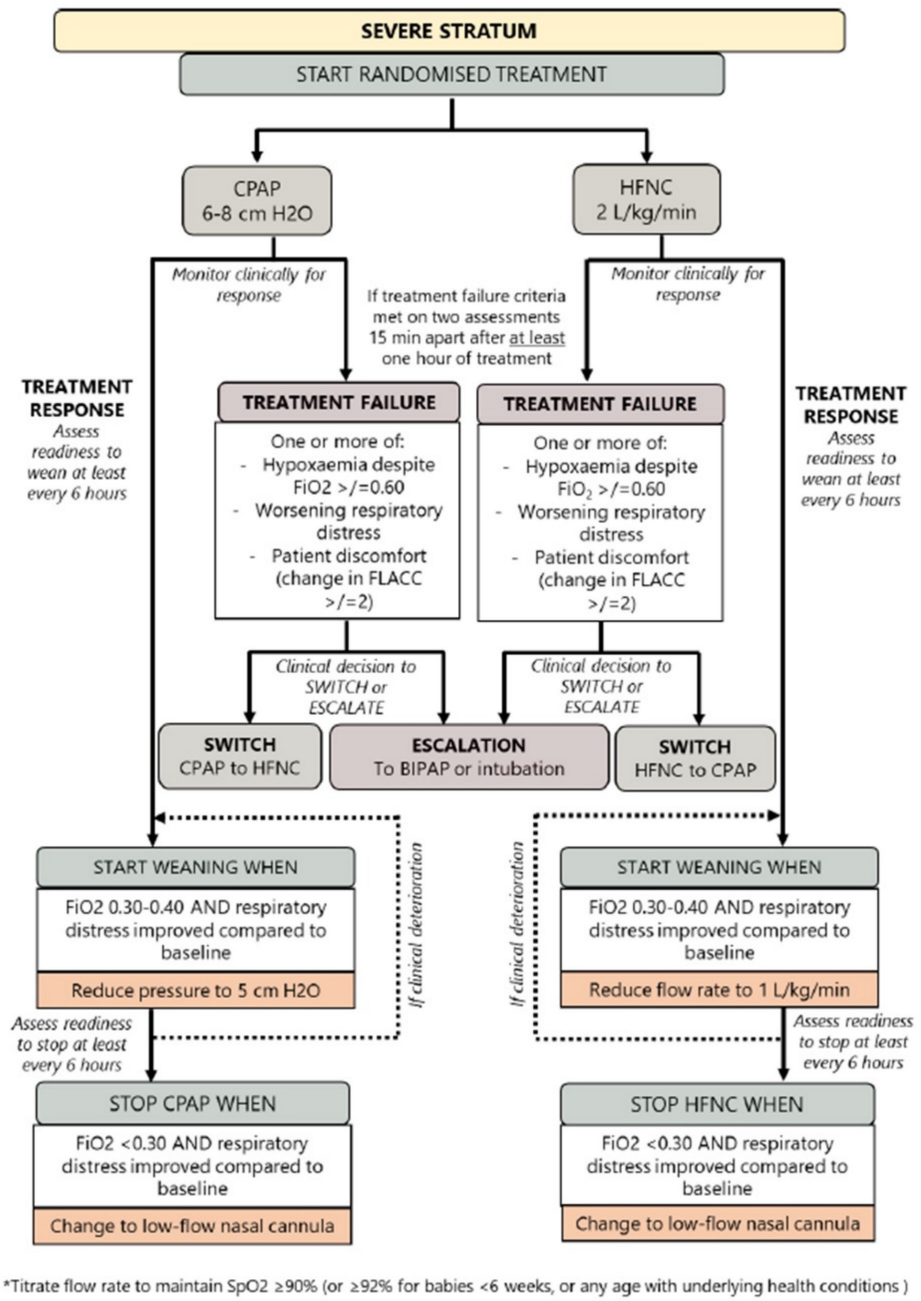
Algorithm for the initiation, weaning, switching, and escalation of treatment for the severe stratum. BIPAP = bilevel positive pressure ventilation, CPAP = continuous positive airway pressure, FLACC = Face, Legs, Activity, Crying, and Consolability, HFNC = high-flow nasal cannula, Spo_2_ = oxygen saturation.

In both strata, to minimize variations in practice, sites will follow study guidance for the commencement, continued delivery and weaning of treatment (**Figs. 1** and **2**). Infants will be assessed for tolerance, response and readiness to wean the assigned treatment at least every 6 hours. A BACHb trial nurse educator will support and train sites to deliver all trial-related treatments.

If patients meet treatment failure criteria, they may be switched to the other arm within their allocated stratum or escalated to a higher form of respiratory support (including intubation) as “rescue treatment.” Patients escalated to bilevel positive pressure ventilation or invasive mechanical ventilation will be treated as per usual site practice (this will not be determined by the trial protocol). All other aspects of care including sedation and feeding for infants receiving the trial treatments will be decided by the treating clinical team.

### Consent Procedures

The BACHb trial will employ a “research without prior consent” (RWPC) model employed in previous trials conducted in pediatric emergency care settings (28–32). Consent will be deferred due to the need to commence urgent treatment and since attempts to obtain informed consent from families during a stressful emergency may compromise patient care. This approach was supported by families during two focus groups conducted as part of the BACHb trial design.

Written consent sought as soon as practically possible (usually within 24–48 hr) after randomization. Consent, using paper-based and electronic methods, will be sought for continued participation in the study, as well as the collection and recording of the child’s clinical data from medical records.

If parents are unable to be approached, or do not consent, before the child’s discharge, the local research team will contact parents by telephone to inform them of their child’s randomization to the study, and to provide study information by post or email (based on parental preference). If there is no response to the call, the local study delivery team will post a covering letter, a copy of the Parent Information Sheet and Informed Consent Form for parents to complete and return to the site. Four weeks after this initial postal contact, another contact attempt will be made stating that if there is no response, the child’s nonidentifiable data collected as part of routine care will be retained. In the rare event where an infant dies before consent has been obtained, a personalized letter will be posted to parents along with the bereaved versions of the consent form and information sheet. The letter will clarify that no further contact will be made, that parents’/legal guardians can refuse consent for their child’s data to be included, and that if there is no response, the child’s nonidentifiable data collected as part of routine care will be retained.

For infants whose parents do not provide informed consent, a minimal amount of data including the child’s month and year of birth will be retained as this will have been collected during the randomization process. However, clinical data after the point of randomization will be not be collected.

### Safety Monitoring

Safety and adverse event (AE) reporting will follow the guidelines of the Health Research Authority (HRA) on safety reporting in studies that do not involve Investigational Medicinal Products. The study protocol categorizes potential AEs of trial treatments which may occur between randomization and hospital discharge: 1) nasal trauma; 2) facial/neck trauma; 3) abdominal distension; 4) pneumothorax; 5) pneumomediastinum; 6) subcutaneous emphysema; 7) facial thermal injury; 8) respiratory arrest; 9) cardiac arrest; and 10) aspiration of gastric contents.

All AEs will be recorded and reported by sites through the OpenClinica system (OpenClinica, Needham, MA) and will be assessed for their severity and relatedness to the trial. Any AEs classified as “severe” or “life threatening” (i.e., “Serious AEs”) will be reported to the trial management teams within 24 hours. Serious AEs (SAEs) determined to be “related” and “unexpected” will be submitted by the Trial Management team to the Yorkshire and The Humber—South Yorkshire Research Ethics Committee (REC) within 15 days of the Chief Investigator being notified of the event.

### Outcome Measures

The primary outcome is the time from randomization to hospital discharge within 30 days. Clinical decision to discharge an infant would usually follow national guidance. Secondary outcome measures are shown in **Table 2**.

**TABLE 2.** Secondary Outcome Measures

**Outcome measure**
1) Proportion of infants experiencing treatment failure
2) Mortality at hospital discharge, day 30, and day 90
3) Proportion of infants requiring intubation and ventilation
4) Proportion of infants requiring admission to an ICU
5) Proportion of infants requiring sedation
6) Duration of oxygen therapy, defined as the time to being free from supplemental oxygen for > 4 hr
7) Time to adequate (75%) oral feeding
8) Time ready for hospital discharge, defined as the time from randomization to latest of time to being free of supplemental oxygen or adequate oral feeding
9) Patient comfort, assessed by the caregiver using the Face, Legs, Activity, Crying, and Consolability scale
10) Patient comfort, assessed by the parent/guardian using a visual analog scale
11) Proportion of infants requiring hospital readmission within 30 d
12) Health status at 30 and 90 d
13) Cost-effectiveness expressed in terms of incremental cost per quality-adjusted life year gained

### Data Collection

**Figure 3.**
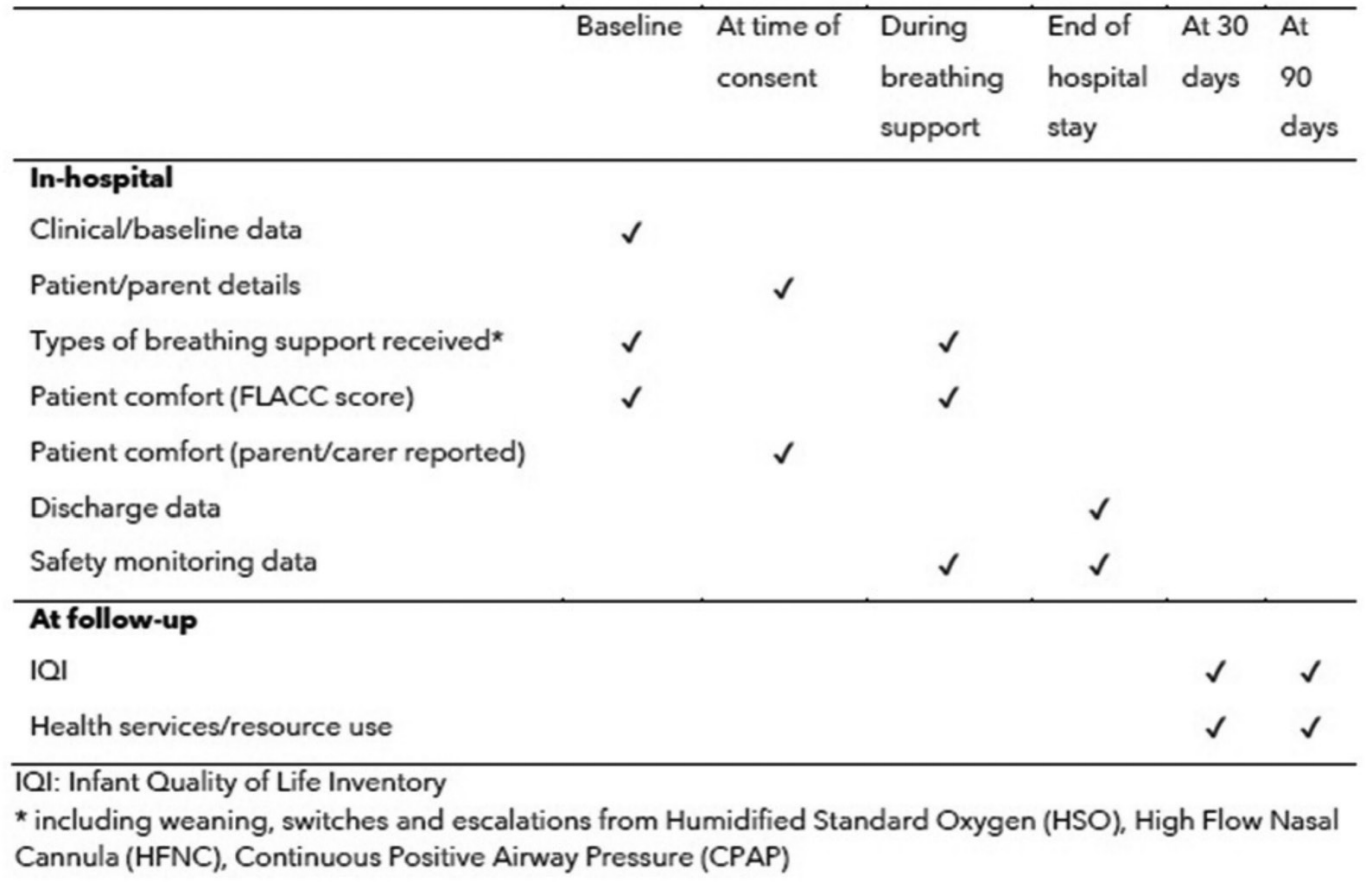
Patient data collection schedule. CPAP = continuous positive airway pressure, FLACC = Face, Legs, Activity, Crying, and Consolability, HFNC = high-flow nasal cannula, HSO = humidified standard oxygen, IQI = Infant Quality of Life Instrument.

Data will be collected and recorded using the electronic OpenClinica system, which is fully validated, records a full audit trail and includes a controlled access system. Sites will enter the required information into the system (**Fig. 3**), which will then be monitored by the Trial Monitor and Trial Manager, including the extraction of reports to monitor the trials progress.

Data collection will include the completion of an Infant Quality of Life Instrument (IQI) questionnaire (33), the Pediatric Quality of Life Inventory and health economic questionnaires to monitor resource usage after discharge at 30 and 90 days post-randomization. These will be sent to parents through either post or electronically and will be entered onto the OpenClinica database.

### Statistical Methods

#### Sample Size

We assumed a median time to discharge of 5 days in the HSO group and 7 days in the CPAP group. The trial aims to detect a 20% reduction in HFNC compared with HSO in moderate bronchiolitis (hazard ratio [HR] = 1.25) and a 25% reduction compared with CPAP in severe bronchiolitis (HR = 1.33). To achieve 90% power at a 5% two-sided significance level, 860 events are required for the moderate group and 527 for the severe group.

Given the low competing risk of mortality (< 2%) and expected discharge within 30 days, we plan to recruit 462 infants per arm (moderate) and 292 per arm (severe), accounting for 7–9% censoring (death or hospital stay > 30 d) and consent refusals. Sample size calculations assume proportional hazards, four sequential tests with Lan-DeMets O’Brien-Fleming spending function. No multiple comparison adjustments are made, as the strata represent distinct treatment comparisons. Calculations were performed using rpact and validated with gsDesign3.3.0 (34, 35).

#### Clinical Effectiveness Analysis

All analyses will be lodged in a statistical analysis plan (SAP), a priori, before the first formal interim analysis. The trial results will be reported according to the Consolidated Standards of Reporting Trials extension for adaptive designs (36). The trial will consist of three formal interim analyses for efficacy when approximately 25%, 50%, and 75% of the planned number of events (hospital discharge) are available. The efficacy boundaries are computed using Lan-DeMets O’Brien-Fleming approximation symmetric efficacy boundaries (37), separately for each stratum (**Supplemental Table 1**, https://links.lww.com/PCC/C650). If the two strata hit the interim target at different time points, decisions for each stratum will be made separately. Statistical reports will be prepared within 1-month of hitting the interim targets. The Data Monitoring Committee (DMC) will review data at the interims and make recommendations to the TSC on whether there are any safety or efficacy reasons to stop the trial. None of the interim analysis results will be shared with the investigators before the completion of the trial.

#### Primary Estimand

The trial aims to determine whether first-line use of HFNC is superior to HSO and CPAP in reducing time to hospital discharge in moderate and severe bronchiolitis, respectively. The primary outcome of time to hospital discharge within 30 days will be measured as days between randomization and hospital discharge or date of last follow-up, whichever is earliest. Deaths will be censored at the time of death, with cause-specific HRs reported under a hypothetical approach for death as an intercurrent event (IE). Other IEs, including treatment escalation, switching, or not starting treatment, will be handled using a treatment policy strategy. Kaplan-Meier plots will display time to discharge distributions, and the Aalen-Johansen estimator will account for death as a competing risk. A cause-specific Cox proportional hazards model, adjusting for age and site, will estimate discharge rates, providing HRs with 95% CIs, with final *p* values judged based on the stage 4 boundary.

#### Supplementary Estimands

Four supplementary estimands will be considered, using a hypothetical strategy for switching and escalation, a principal stratum approach for not starting the allocated treatment or any respiratory support, and a composite strategy incorporating the Fine and Gray model for death. Details of the supplementary estimand analyses will be provided in the SAP.

#### Secondary Outcomes Analysis

Binary outcomes, such as mortality at hospital discharge, will be analyzed using a log-binomial model with a fixed effect for age (< 6 and ≥ 6 wk) and a random effect for study site (as a random intercept) to estimate an adjusted risk ratio with a 95% CI and *p* value. For continuous outcomes measured at a single time point, a linear mixed model with a fixed effect for age and a random effect for study site will be used. An adjusted mean difference with a 95% CI and *p* value will be reported.

Longitudinal continuous outcomes will be analyzed using a mixed-effects model to estimate the average treatment effect over time. Time-to-event outcomes will be visualized with Kaplan-Meier plots, with HRs and 95% CIs estimated using the primary analysis model.

#### Subgroup Analyses

Interaction effects between treatment and the following factors will be assessed: age groups, preterm infants (< 37 wk), prior bronchiolitis admission, underlying conditions, hospitals with on-site PICU, and respiratory syncitial virus (RSV) diagnosis. The study is not powered to detect interaction effects. Interaction effects will therefore be judged interesting with *p* values of less than 0.2.

#### Safety Analysis

AEs will be coded using Medical Dictionary for Regulatory Activities and summarized by severity, seriousness, and relatedness to treatment within each stratum. SAEs and Adverse Events of Special Interest will be reported in detail. The number of participants with at least one AE, the mean event count per participant, and occurence rates will be presented. If AE counts are sufficiently large, odds ratios and incidence rate ratios with 95% CIs will be estimated using appropriate regression models. Dot plots and horizontal stacked bar charts will visualize AE distributions and severity by treatment arm.

#### Health Economics Evaluation

The trial design includes an integrated prospective economic evaluation conducted from an National Health Service (NHS) and personal social services perspective. The economic evaluation will compare HSO vs. HFNC and HFNC vs. CPAP by understanding the use of resources and health-related quality of life outcomes over 90 days post-randomization. The measurement of these resources and outcomes will be captured on trial case report forms, as well as through the questionnaires completed by parents after discharge. Quality-adjusted life years (QALYs) profiles will be generated through the completion of the IQI (38, 39). The incremental cost per QALY gained by each treatment modality will be compared within each stratum to evaluate their cost-effectiveness. Areas of uncertainty regarding components of the economic evaluation will be assessed through sensitivity analyses, which will include adopting a societal perspective that captures direct costs borne to families and economic values for informal care and productivity losses. The probability of the cost-effectiveness of HSO vs. HFNC and HFNC vs. CPAP will be displayed on cost-effectiveness acceptability curves.

### Ethics and Patient/Public Involvement

#### Research Ethics

The Yorkshire and The Humber—South Yorkshire REC and HRA provided approval for the BACHb trial (REC reference: 23/YH/0166, IRAS: 327621, study title: Breathing Assistance in CHildren with bronchiolitis (BACHb): a group-sequential two-stratum multicentre open-label randomised clinical trial of respiratory support in infants with acute bronchiolitis, approval date: August 3, 2023). Each participating NHS Trust must also provide its own confirmation of capacity and capability before study delivery may commence at the site. Study procedures were followed in accordance with U.K. HRA ethical standards and with the Helsinki Declaration of 1975.

#### Confidentiality

Confidentiality will be maintained at all stages of the trial as per the requirements of Data Protection Act (2018) including in the collection, storage, processing, and disclosure of personal information. Data collected will be pseudonymized, stored securely, and will only be accessed by staff who have undergone ICH-GCP and data protection training.

#### Patient and Public Involvement

Two patient and public involvement (PPI) focus groups were conducted during trial development, one face-to-face and one online, which provided valuable insights regarding trial design and methods, particularly regarding use of the RWPC model. Members supported the need for research into the treatment of pediatric respiratory support. Two members of the PPI group are included as a part of the Trial Management Group (TMG). Additionally, an independent parent advisory group was established with six parents/carers who will meet annually to advise on any changes needed during the trial.

### Governance and Oversight

While the TMG will oversee the trial’s day-to-day management through the Imperial Clinical Trials Unit, the TSC will provide overall supervision. The DMC will review interim analyses and advise the TSC, including on safety events affecting trial continuation. Imperial College London sponsors the trial (reference: 22HH7629) and holds indemnity and insurance for legal liability related to its design, management, and conduct.

### Trial Status

BACHb is an ongoing multicenter trial. This article presents the trial protocol version 3.0 dated August 21, 2024 (accessible at bachbtrial.org.uk). The first patient was recruited in October 2023. After the internal pilot phase, the study was assessed by the TSC and the funder and was supported to continue to the full trial. Patient recruitment is ongoing with recruitment planned to end March 2026. Partway through the trial (September 2024), a national vaccination program for pregnant women was introduced to protect infants from severe RSV infection, which may reduce the number of eligible children. However, vaccine uptake is increasing slowly over time, and we do not expect this to significantly affect patient recruitment within the trial’s timeline. Trial findings will be disseminated through publication in peer-reviewed medical journals and at national and international conferences as well as social media and lay channels.

## Supplementary Material


