# Supplementary material for: Protocol for a Group-Sequential Two-Stratum Multicenter Open-Label Randomized Clinical Trial of Respiratory Support in Infants With Acute Bronchiolitis: Breathing Assistance in Children With Bronchiolitis (BACHb)
Source: Pediatr Crit Care Med. 2025 Aug 14;26(10):e1275–85. doi: 10.1097/PCC.0000000000003813 (PMC12502951; doi:10.1097/PCC.0000000000003813)
Supplement: Supplementary file 1 [file pcc-26-e1275-s001.docx]

Supplemental Table 1: Efficacy boundaries for the trial interim analyses

|  | **Moderate population** | | | | **Severe population** | | | |
| --- | --- | --- | --- | --- | --- | --- | --- | --- |
| Stage | I | II | III | IV | I | II | III | IV |
| % of total  Number of events at Interim | 25% | 50% | 75% | 100% | 25% | 50% | 75% | 100% |
| Events across both arms | ≈ 215 | ≈ 430 | ≈ 645 | ≈ 860 | ≈ 132 | ≈ 264 | ≈ 395 | ≈ 527 |
| Z score for  two-sided  efficacy  boundaries | -4.33 and 4.33 | -2.96 and 2.96 | -2.36 and 2.36 | -2.01 and 2.01 | -4.33 and 4.33 | -2.96 and 2.96 | -2.36 and 2.36 | -2.01 and 2.01 |
| P-value  boundaries | 0.00001 | 0.00305 | 0.01832 | 0.04400 | 0.00001 | 0.00305 | 0.01832 | 0.04400 |
